# Metabolic and ultrastructural renal changes in adult Wistar rats fed by a cafeteria diet

**DOI:** 10.1590/acb392224

**Published:** 2024-04-15

**Authors:** Priscila Fernandes dos Santos, Diogo Benchimol de Souza, Eduardo José Lopes Torres, Waldemar Silva Costa, Francisco José Barcellos Sampaio, Bianca Martins Gregorio

**Affiliations:** 1 Universidade do Estado do Rio de Janeiro – Biomedical Center – Department of Anatomy – Rio de Janeiro (RJ) – Brazil.; 2 Universidade do Estado do Rio de Janeiro – Biomedical Center – Department of Microbiology, Immunology and Parasitology – Rio de Janeiro (RJ) – Brazil.

**Keywords:** Diet, Rats, Microscopy, Electron, Scanning, Microscopy, Electron, Transmission

## Abstract

**Purpose:**

To evaluate, by quantitative and qualitative methods, the glomerular ultrastructure in Wistar rats fed a cafeteria diet.

**Methods:**

Male Wistar rats were divided into two groups at 21 days of age: control (C, n = 10) and cafeteria diet (CAF, n = 8). The animals were followed up until 5 months of age, followed by euthanasia. The blood, kidneys, and fat deposits––epididymal, retroperitoneal, and subcutaneous––were extracted and analyzed. Data were analyzed by Student’s t test, and p < 0.05 was considered statistically significant.

**Results:**

The cafeteria diet promoted glucose intolerance, hyperglycemia (p < 0.0001), and deposition of retroperitoneal fat (p < 0.005). Scanning electron microscopy revealed that the length of the foot process was similar in both groups. The quantitative analyses by transmission electron microscopy revealed that the cafeteria diet reduced the thickness of the glomerular basement membrane (p < 0.05).

**Conclusions:**

The intake of lipids and simple carbohydrates were found to be associated with alteration in the glomerular ultrastructure. However, more studies are needed to evaluate not only the effects of high-protein and high-fat diets on components of the glomerular filtration barrier, but also renal physiology.

## Introduction

Dietary patterns can be analyzed to elucidate the relationship between diet and health problems. Scientific studies show that eating patterns are quite variable among the people. The consumption of vegetables, fruits, fiber, and fish is beneficial for human health, while the intake of red meat and processed foods is harmful^1^. In addition to the body modifications promoted by this nutritional imbalance, an increase in the development of chronic diseases, such as overweight / obesity, diabetes mellitus, hypertension, and chronic kidney disease has been observed^2^.

Research involving experimental models and nutrition has been widely carried out in order to elucidate the pathophysiology of several non-transmittable chronic degenerative diseases. Among these diets is cafeteria diet, which is a high source of energy and palatable^3^. Its lipid and simple carbohydrate content favors the development of glucose intolerance, besides causing morphological changes in the reproductive system, increase of body mass and abdominal fat, hyperinsulinemia, and hyperglycemia^3^^,^ 4. In animals, the intake of cafeteria diet can trigger morphological changes in kidneys, such as interstitial fibrosis and tubular atrophy^5^. However, the influence of this diet on the renal ultrastructure has not yet been elucidated.

It is already known that high-protein diets can dilate the afferent glomerular arteriole, resulting in hyperfiltration and subsequent glomerular damage (inflammation and fibrosis)^6^. Similarly, high-fat diets alter renal physiology, generating glomerular hypertrophy, proteinuria, increased desmin expression, and reduced nephrine expression in the glomerulus^7^. The glomerular filtration barrier is extremely important in the filtration process of the plasma. It consists of a fenestrated glomerular endothelium^8^, supported by a basement membrane^9^.

In this study, we examined specialized epithelial cells of the glomerular filtration barrier (podocytes) with their long cellular processes involving the blood capillaries (foot processes). Therefore, the detailed study of this component, in view of the various macronutrients present in our diet, is necessary, considering the increase in the consumption of such foods of low nutritional value (rich in lipids and simple carbohydrates) in society.

The aim of this study was to evaluate the glomerular ultrastructure, especially the podocytes and the glomerular basement membrane, of rats fed with cafeteria diet.

## Methods

### Experimental animal procedures

All procedures were performed with the approval of the Universidade do Estado do Rio de Janeiro Animal Care and Use Committee. Male Wistar rats (n = 18) were obtained from Urogenital Research Unit and housed in cages at 21 ± 2°C with a 12-h light/dark cycle. At weaning, the animals were randomly selected to receive either control diet (C, n = 10) or a cafeteria diet (CAF, n = 8) up to 5 months of age, followed by euthanasia. Both groups were fed *ad libitum* with fresh food daily. C diet consisted of commercial food (Nuvilab)–430 kcal/100 g–, and CAF diet was manipulated in the laboratory, having following constituents: commercial food 60 g/100 g, condensed milk (Nestlé) 25 g/100 g, and hydrogenated vegetable fat (Primor) 15 g/100 g, totaling 550 kcal/100 g. Food intake and body mass were assessed daily and weekly, respectively.

After being fed for 20 weeks with the assigned diet, rats were fasted for 12 h, anaesthetized with 100 mg/kg of sodium pentobarbital and killed, with blood taken directly from the right atrium. After collection, the blood was centrifuged, and plasma was obtained. The kidney and the different white adipose tissue depots (epididymal, retroperitoneal, and subcutaneous) were removed, weighed, and fixed until further analysis. Fat deposits were fixed in buffered formalin (4%) for 48 h. After this period, these tissues were processed, and 5-μm thick sections were cut.

### Carbohydrate metabolism and biochemical assays

The oral glucose tolerance test was performed at 5 months of age. Following an overnight fast (12 h), a baseline blood glucose level was measured, and then all animals were administered a dose of hypertonic glucose serum 50% (2 g/kg body weight) by orogastric gavage. Blood glucose concentrations were measured 15, 30, 60, and 120 min after the glucose administration by glucometer (Accu-Chek, Roche, São Paulo, SP, Brazil).

Moreover, plasma glucose, triacylglycerol (TAG), high-density lipoprotein (HDL-C), and total cholesterol (TC) levels were measured using enzymatic colorimetric kits (BioSystems–Cat. 11506–Barcelona, Spain). Insulin levels were analyzed with a rat insulin enzyme-linked immunosorbent assay (ELISA) kit (Millipore–Cat. EZRMI-13K–St. Charles, Missouri, United States of America), according to the manufacturer’s protocol.

### Kidney collection and morphometry

For scanning electron microscopy (SEM), the kidney was removed, and fragments of approximately 1 mm^3^ were collected from the cortical region. These fragments were fixed by immersion in glutaraldehyde (2.5% in phosphate buffer, pH = 7.3) during 24 h and post fixed with 1% osmium tetroxide and 0.8% potassium ferrocyanide for 40 min at room temperature. Then, they were dehydrated in ethanol, critical point-dried with CO2, sputter-coated with gold-palladium, and observed using a Auriga Compact SEM at a magnification of approximately 27,000x and with a 2 kV beam acceleration voltage.

For transmission electron microscopy (TEM), after post fixation, the fragments were dehydrated in acetone and embedded in Epon. Ultra-thin sections (50 nm) were obtained and contrasted with 1% lead citrate and 5% uranyl acetate. The sections were observed on a JEOL-JEM-1011 TEM of the Rudolf Barth Electronic Microscopy Platform (Fundação Oswaldo Cruz), at a magnification of 50,000x and beam acceleration voltage of 80 kV.

SEM images were used to measure the length of the foot process, while in TEM images the thickness of the glomerular basement membrane (GBM) and the linear length of the foot process in contact with the GBM were measured. For both microscopy techniques, five animals/group and an ImageJ Software (Image Processing and Analysis in Java) were used.

### Statistical analysis

Values were expressed as mean ± standard deviation. Student’s t test was used to compare the two groups using GraphPad Prism 5. In all cases, p < 0.05 was considered as statistically significant.

## Results

Food intake was similar between the groups. Corroborating with this result, the groups showed no difference in weight gain throughout the experiment. However, the CAF diet increased the deposits of retroperitoneal fat when compared to the control (Table 1).

**Table 1:** Biometric and kidney data.

Parameters	C	CAF	p-value
Food intake (g)	8.24 ± 0.46	6.98 ± 0.54	0.0943
Weight gain (g)	350.20 ± 25.72	350.40 ± 39.84	0.9867
Retroperitoneal fat (g)	5.02 ± 1.26	8.91 ± 3.40 a	0.0036
Subcutaneous fat (g)	3.14 ± 0.71	4.37 ± 1.38	0.0626
Epididymal fat (g)	5.43 ± 1.15	7.41 ± 1.77	0.0750
OGTT After-resveratrol (u.a.)	745.00 ± 42.84	869.50 ± 67.21a	< 0.0001
Serum glucose (mmol/L)	10.47 ± 4.32	16.22 ± 5.62a	0.0361
Cholesterol total (mg/dL)	59.89 ± 5.64	60.75 ± 8.01	0.7995
Triacylglycerol (mg/dL)	85.60 ± 29.51	103.80 ± 38.69	0.2625
High-density lipoprotein (mg/dL)	38.90 ± 7.01	40.44 ± 7.00	0.6376
Insulin (ng/mL)	3.92 ± 1.58	6.37 ± 0.75	> 0.05
Foot process lenght (µm)	0.19 ± 0.09	0.14 ± 0.06	0.2200
Glomerular basement membrane thickness (nm)	147.40 ± 10.41	126.50 ± 6.76a	< 0.0001
Length of foot process/glomerular basement membrane segment (nm)	328.30 ± 30.50	273.60 ± 12.69	0.1359

Data were presented as mean ± standard deviation. Differences were tested by Student’s t test; p < 0.05; ^a^statistical difference for the C group.

Source: Elaborated by the authors.

The cafeteria diet promoted glucose intolerance and hyperglycemia at 5 months of age. The CAF group showed an elevation in the area under the glucose curve (↑17%) when compared to C group (p < 0.0001). Moreover, serum glucose levels remained significantly higher in the CAF group compared to the control. However, cafeteria diet was not able to significantly modify the serum levels of TC, HDL-C, TAG, and insulin of the CAF group compared to the control group (Table 1).

**Figure 1:**
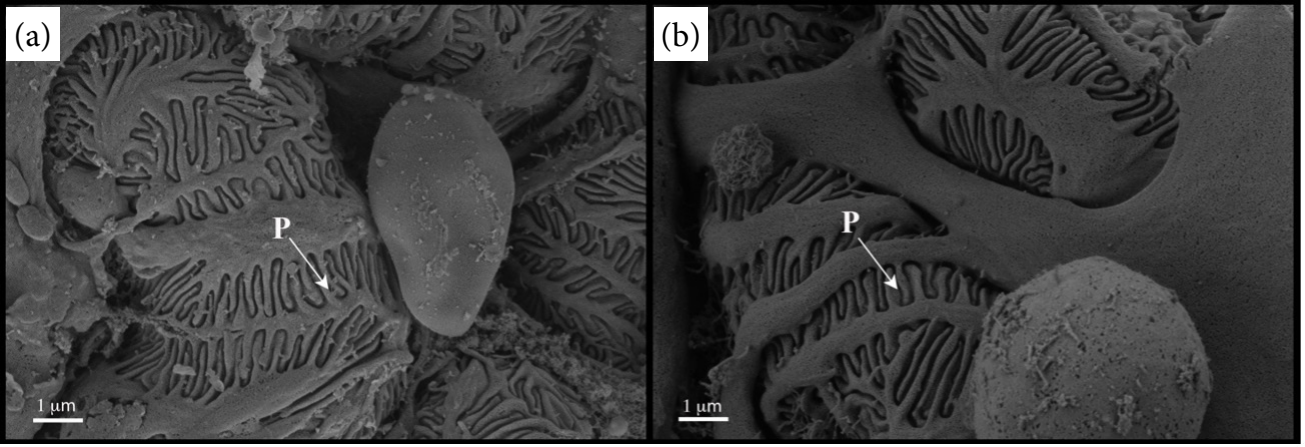
Scanning electron Microscopy images of glomerulus. We observed that the lengths of foot processes **(P)** were similar between animals from **(a)** control and **(b)** cafeteria diet groups. Both images were obtained at the magnification of 27,000 (approximately) and a beam acceleration voltage of 2 kV.

Source: Elaborated by the authors.

The qualitative analysis of the length of the foot process by SEM revealed it to be comparable between the groups studied (Fig. 1), which was corroborated by the statistical analysis (Table 1).

**Figure 2:**
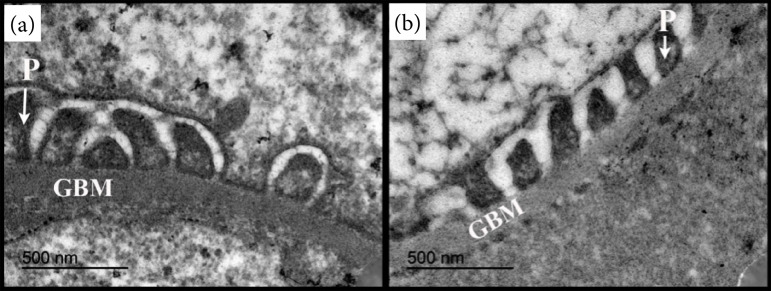
Transmission electron microscopy images of glomerular filtration barrier components: glomerular basement membrane (GBM) and foot processes **(P)**. **(a)** Control group: the GBM thickness is higher when compared to the animals fed with **(b)** cafeteria diet. Cafeteria diet also led to the reduction in the foot process length **(P)** compared to the control group. Both images were obtained at the magnification of 50,000 and a beam acceleration voltage of 80 kV.

Source: Elaborated by the authors.

TEM measurements showed a marked reduction of GBM thickness in the CAF group (Fig. 2) and tendency to reduce the length of foot process by GBM segment when compared to control (Table 1).

## Discussion

The intake of cafeteria diets has previously been correlated with obesity^8^^,^ 9 and important morphological changes in the rodent kidneys, such as glomerulosclerosis5, glomerular hypertrophy followed by excess mesangial matrix^10^^,^ 11, and glomerular hyperfiltration^12^. In our study, similar to the findings of Gomez-Smith et al.^13^ and Higa et al.^14^, cafeteria diet did not promote an increase in the body mass of the animals, although it caused glucose intolerance and an elevation of the retroperitoneal fat deposits.

The exacerbated consumption of foods rich in simple carbohydrates and lipids, together with sedentary lifestyle, promotes hypertrophy of adipose tissue, mainly the visceral fat deposit^15^^,^ 16. The visceral adiposity accentuates lipolysis and leads to increased release of free fatty acids from adipose tissue. Thus, there are reduction in glucose mobilization and a greater risk of the development of glucose intolerance and insulin resistance, which justify our findings^17^^,^ 18. The limitations of this study were the use of rats and a diet with low lipid content, which was not indicative of variation in body mass.

In relation to the kidney, excessive visceral adiposity in humans promotes renal compression, with increased intrarenal pressure^19^^–^ 21, and is associated with coronary artery calcification in patients with chronic kidney disease^22^. However, we did not find the presence of hypertension in the CAF group. It is possible that the physical compression of the kidneys in humans, rabbits, and dogs is different from that in rodents. Anatomically, the kidneys of these mammals appear to float more than adipose tissue and, therefore, are not compressed, preventing the reduction of renal blood inflow and maintaining the blood pressure at normal levels^21^^,^ 23.

The redistribution of body fat caused by intake of the cafeteria diet may also affect the renal ultrastructure^24^. Previous studies indicated that lipotoxicity leads to changes in the cytoskeleton of podocytes and in the proteins of the slit diaphragm^25^. Similarly, the toxicity mediated by saturated fatty acids, such as palmitic acid, induces endoplasmic reticulum stress and podocyte death^26^. In our study, the increase in retroperitoneal fat in the CAF group did not promote significant alterations in this structure. Our qualitative and quantitative analyses by SEM and TEM, respectively, showed that the cafeteria diet caused a slight reduction in podocyte length (not significant). It has been assumed that, at this stage, this diet promoted a remodeling of the podocyte cytoskeleton, neutralizing renal injury^27^.

The podocytes are differentiated cells that cover the outer surface of the GBM, and contain three main segments: the cell body, the primary processes, and the foot process, and they are indispensable for the normal maintenance of glomerular filtration. Previous literature reports that high-protein and high-fat diets (without the association of sucrose) promote podocyte effacement, with apparent impairment of renal function^7^^,^ 28. However, in the present study, reduction of GBM thickness was verified in the CAF group, regardless of the reduction of foot process length. It is believed that sucrose, the main macronutrient present in the diet used in this study, is not involved neither in the initiation and progression of glomerulosclerosis nor, consequently, in the manifestation of renal injury.

## Conclusion

It was noticed that macronutrients, except sucrose, are directly associated to the loss of renal function. Even though we did not work with diets rich in proteins and lipids, we saw that simple carbohydrates modified only the GBM thickness, without compromising the length of the foot process. However, more studies are needed to evaluate not only GBM components, but also renal physiology.

## Data Availability

All data sets were generated or analyzed in the current study.
